# The Correlates of Academic Stress in Hong Kong

**DOI:** 10.3390/ijerph19074009

**Published:** 2022-03-28

**Authors:** Esther Pui Yung Chyu, Ji-Kang Chen

**Affiliations:** Department of Social Work, The Chinese University of Hong Kong, Hong Kong; jkchen@swk.cuhk.edu.hk

**Keywords:** academic stress, East Asia, perfectionism, social-oriented achievement motivation, parental aspiration for achievement, parent–child relationship, emphasis on academics in schools, school climate

## Abstract

Most previous studies have attempted to explore how different personal, familial, or school factors are linked to academic stress in Western countries. However, relatively less research has incorporated these different factors into one model to examine the most crucial correlate(s) that predict academic stress, particularly in the East Asian context, where the level of academic stress among adolescents is high. This study examined how perfectionism, social-oriented achievement motivation, parental aspiration for achievement, parent–child relationship, emphasis on academics in school, and school climate work together to predict academic stress in Hong Kong. One thousand eight hundred and four students from eight secondary schools in Hong Kong participated in this study. The results indicate that perfectionism, social-oriented achievement motivation, parent–child relationships, and emphasis on academics in school have significant associations with academic stress, while perfectionism and social-oriented achievement motivation, the two factors from the personal domain, are the dominant drivers of academic stress. In addition, these findings applied to both genders. As the significant correlates come from the personal, familial, and school domains, this study recommends multilevel interventions for decreasing the level of academic stress. In addition, this study also suggests further research directions to examine the psychosocial mechanism between the correlates and academic stress.

## 1. Introduction

Academic stress poses an increasingly widespread threat to young generations, irrespective of their age groups, gender, or academic performance. Research has indicated that overwhelming academic stress is strongly associated with poor academic performance and procrastination [1,2], physical illness [3,4,5], mental distress [6,7,8,9], and suicidal ideation and attempts [10,11]. For that reason, understanding the correlates of academic stress is imperative for designing helpful preventive interventions to mitigate the harmful effects of academic stress. Researchers have identified an extensive list of correlates that may be associated with academic stress. For example, theories about personality suggest that personal factors, such as perfectionism and endorsing social-oriented achievement motivation [2,12,13], are critical determinants of academic stress [14,15,16,17]. In addition, theories and models pertaining to families argue that the parent–child relationship and parental aspirations for achievement are significant factors predicting academic stress [12,18,19,20]. In addition, other theories related to the school context, such as social setting theory and stage-environment fit theory, propose that the school climate and how schools emphasize academics are important factors that may affect adolescents’ school adjustment and academic stress [21,22,23,24]. These correlates are identified from different domains or social contexts that are significant to adolescents, including the school, family, and personal domains. However, most of these studies examined the association between these correlates and academic stress separately, and in Western contexts, it is not clear what factor(s) are more significant in predicting academic stress, as less research has incorporated different factors into one model. In addition, it is also not clear whether the associations between the correlates and academic stress in Western contexts are also manifested in East Asian contexts, where they are found to have a high prevalence of academic stress [25,26]. Taking these concerns into consideration, the current study incorporated different factors from the personal, familial, and school domains into one model and investigated the significant correlates that trigger academic stress in the East Asian context. Furthermore, this study also examined whether the theoretical model was applicable to both males and females. The empirical findings of this research can not only advance our understanding of the risk factors for academic stress but also guide policy makers in allocating resources and facilitating the development of appropriate preventive interventions addressing academic stress for adolescents.

## 2. Literature Review

### 2.1. Personal Factors Associated with Academic Stress

Theories and empirical studies have argued that personal factors are critical determinants of academic stress [14,15,16,17]. For example, the transactional model of stress posits that individual cognitive appraisal affects how a person evaluates and perceives a potential threat, and different people react differently in similar stressful environments [27]. If an individual perceives that the demands outweigh the resources, that individual may experience stress [27]. In addition, personality psychology theories state that individual personalities may affect evaluative appraisal and trigger emotional reactivity, which in turn may lead to an increase in the level of stress, including academic stress [15,28]. Among the personal factors, perfectionism and endorsement of social-oriented achievement motivation have been most often discussed in the literature in terms of their association with academic stress [2,12,13].

Perfectionism: Perfectionism is a performance-related personality characteristic that may be linked to academic stress [2]. Personality psychology posits that certain personality traits, including perfectionism, are associated with increased exposure to stress [29,30]. Specifically, perfectionists may create and impose stress on themselves by placing themselves in high-pressure situations and then negatively evaluating their performance in such situations [2,29]. Several empirical studies have demonstrated that people with maladaptive perfectionism tend to have extraordinary academic standards and persistent self-criticism. These traits may lead to disappointment and self-doubt in their academic performance, and self-condemnation and frustration can then trigger academic stress [2,28,31]. Although the association between perfectionism and academic stress has been shown in several empirical studies [2,31], most of those studies were conducted in Western cultures and have targeted undergraduates or high achievers as their sample subjects [2,31]. In most East Asian countries, high school students have to take public examinations upon graduation. These public examinations are usually high-stakes assessments because the results of the examinations have substantial implications for their future trajectory. Consequently, high school adolescents have comparatively greater chances of experiencing academic stress. However, relatively few studies have been conducted to investigate whether perfectionism predicts academic stress in high school students in East Asian societies. Based on the literature on perfectionism and other previous empirical studies, this study hypothesizes that perfectionism is positively associated with academic stress.

Social-oriented achievement motivation refers to a motivational system that is driven by a desire to gain social approval from significant others and bring honor to one’s family [32]. Specifically, people who endorse social-oriented achievement motivation will pursue achievement goals and standards of excellence set by significant others [33]. They will also evaluate their success or failure against the validation and approval they receive from their significant others. Thus, social-oriented achievement motivation reflects high social instrumentality and weak functional autonomy, in contrast with individual-oriented achievement motivation, which is fueled by a desire to attain one’s own aspirations and to realize one’s talents [32].

Academic achievement has different meanings, depending on the sociocultural context of society [13]. In Western societies, academic achievement is regarded as an individual endeavor, and people are encouraged to pursue goals that meet their own needs and preferences [34]. In contrast, in East Asian cultures, academic achievement is seen as a social endeavor [34], in which individual achievement is not only a person’s own quest for knowledge but also a means of bringing honor and fame to the family [35]. To date, there has been only one study suggesting that students with social-oriented achievement motivation will experience examination stress because they are worried about how their significant others will evaluate their examination performance [13]. However, the participants in that study were college students, and the dependent variable was examination stress rather than academic stress. Although examination stress and academic stress are sometimes interchangeable in the literature, their definitions are different [36]. Examination stress refers to the specific pressures induced by examinations and assessments, while academic stress denotes general anxiety and feelings of stress about studying and academia [36]. In sum, little is known about whether endorsing social-oriented achievement motivation predicts academic stress in high school students in the East Asian context. Based on the literature and empirical studies, this study hypothesizes that the endorsement of social-oriented achievement motivation is positively associated with academic stress.

### 2.2. Familial Factors Associated with Academic Stress

Family theories have argued that familial factors and contexts are crucial influencers of behavioral, emotional, and academic outcomes in youth [12,18,19,20]. For example, the family process model suggests that economic stress in family life and couple relationships would impair parents’ well-being, weaken parent–child relationships, and arouse intergeneration conflict, which would adversely affect children’s behavioral, emotional, and academic adjustment [20,37]. In addition, from the perspective of attachment theory, the quality of the parent–child relationship and the responsiveness of parents will affect the bonding built between the parents and the children, which will in turn influence how the children develop their personality, cognitive, social, and emotional outcomes through an internal working model [38,39,40]. In sum, these theories highlight the importance of familial experiences and contexts in affecting adolescents’ well-being and academic adjustment through daily communication. Among the familial factors, the parent–child relationship and parental achievement aspiration have been found to be associated with children’s academic stress [18,19,41,42,43].

The parent–child relationship refers to the emotional ties between parents and offspring. In the literature, the parent–child relationship has been found to be a significant factor supporting the child when the child faces challenges in the social environment [40]. An intimate and positive parent–child relationship is related to fewer externalized and internalized problems among adolescents [44,45]. Empirical findings have suggested that there is a significant association between the parent–child relationship and academic stress [45,46,47]. However, the direction of the link between the parent–child relationship and academic stress is opposite in Western and Eastern societies. In the Western literature, the parent–child relationship has been found to have a negative association with academic stress [44,45]. When students gain a sense of security from intimate parent–child relationships, they can freely express their fears and worries regarding learning. Such open and sincere communication can facilitate their search for solutions to their learning difficulties, helping them reduce their level of academic stress. However, other studies conducted in Asian societies have suggested the opposite, i.e., a closer parent–child relationship is associated with a higher level of academic stress [18,41]. Cultural studies have suggested that Chinese families have high expectations of children’s academic achievements [48,49]. Good rapport with their parents may induce children to adhere more to their parents’ academic goals and demands, causing the children to have a greater sense of an obligation to fulfill their parents’ high academic expectations, which in turn triggers more academic stress [18,50]. Although a positive association between the parent–child relationship and academic stress has been shown in primary school students in East Asian societies, little is known about whether such a positive association is also found in high school students. Based on the theoretical literature and the findings of empirical studies, this study hypothesizes that the parent–child relationship is positively associated with academic stress.

Parental achievement aspiration refers to the emphasis that parents place on the importance of achievement and accomplishment [19,42]. Such achievement aspirations have been found to be a common feature in East Asian societies [10,48], such as those in Korea, Japan, Taiwan, Singapore, and Hong Kong. All these societies have been deeply influenced by Chinese culture, in which academic success is considered to be not only a means of personal achievement but also a way of fulfilling one’s familial obligations [35,51,52]. Several empirical studies have suggested that perceived parental achievement aspiration is linked to the level of academic stress [19,42]. For example, a study has shown that Hong Kong adolescents’ perceived parental achievement pressure imposes a substantial burden on them, especially when the adolescents are aware that academic achievements please their parents [53]. Other empirical studies have also indicated that parental attitudes toward academic attainment are related to students’ levels of academic stress [42,54,55]. Although parental achievement aspirations are common in East Asian societies, relatively few studies have examined how parental achievement aspiration is associated with academic stress in high school students in the East Asian context. Based on the literature, this study hypothesizes that perceived parental achievement aspiration is positively associated with academic stress.

### 2.3. School Factors Associated with Academic Stress

Theories and models related to the school context have suggested that the school environment is a significant factor affecting adolescents’ well-being and academic adjustment, including academic stress [21,22,23,24]. For example, the social setting theory suggests that the school environment plays a crucial role in adolescents’ mental well-being [56]; the stage-environment fit theory posits that school contexts, such as the learning atmosphere and classmate interactions, are crucial factors affecting students’ academic performance, school engagement, mental health, and education-related stress [57,58,59]. Eccles and her research team argue that an unfavorable school environment, signifying a mismatch between the needs of adolescents and the opportunities provided by their education environment, would probably lead to maladjustment and academic-related problems, such as school drop-out, lack of learning motivation and test stress [57,58]. Among the school factors, school climate and emphasis on academics are speculated to be associated with academic stress [58,60,61,62].

School climate is based on the patterns of students’ experiences of school life and reflects the norms, goals, values, interpersonal relationships, teaching and learning practices, and organizational structures [63]. In addition, school climate is also a construct that encompasses the different aspects of the school environment, such as peer interactions, relationships with teachers, and the classroom atmosphere [60]. Extensive empirical studies on school climate have suggested that the school environment plays a crucial role in students’ outcomes, including academic performance, behavioral appearance, psychosocial well-being, and educational stress [60,62,64,65,66]. Researchers have argued that a nurturing school climate can promote a sense of belonging and eagerness to study and participate, which can in turn promote psychological well-being [58,60]. In contrast, a negative school climate discourages learning and leads to the development of uncomfortable feelings; such an unfavorable school environment, which results from a mismatch between the needs of adolescents and the opportunities provided by their school environment, will probably lead to maladjustment and academic-related problems, such as school drop-out, a lack of motivation to learn, and examination stress [57,58]. Although the association between school climate and academic stress has been shown in Western societies, few empirical studies have been conducted to examine the link between school climate and academic stress in East Asian societies. Asian researchers have argued that the school environment and climate differ between Eastern and Western cultures due to variations in the curriculum, class size, and teacher–student ratio [67]. Based on the theoretical literature and the findings of empirical studies, this study hypothesizes that school climate is negatively associated with academic stress.

Emphasis on academics in school refers to the degree to which forces imposed by the school environment exert pressure on student academic performance [68]. These forces also refer to the behavioral expectations of the students, which include working diligently in school so they can attain specific academic standards [68,69]. This behavioral expectation emphasizes the assumptions that high standards bring about greater efforts and that greater efforts lead to greater achievement [69]. However, researchers have argued that such academic emphasis does not always lead to positive outcomes [68,69]. The findings of empirical studies have suggested that the emphasis on academics in schools, imposed by demands, persuasion, and comparisons, may result in detrimental mental problems in adolescents, such as depression, a sense of inadequacy, and academic stress [62,70]. Although an emphasis on academics is commonly found in East Asian schools, little is known about whether this emphasis in schools is associated with academic stress in high school students in the East Asian context. Based on the theoretical literature and empirical studies, this study hypothesizes that perceived academic pressure is positively associated with academic stress.

### 2.4. Interactions among Factors and Their Effects on Academic Stress

Previous studies have identified the abovementioned correlates of academic stress. These factors are either related to personal characteristics or can be found in the school or familial domain. However, these studies have not informed us on which factor is the most crucial in leading to academic stress, because most of them have only examined the associations between these correlates and academic stress separately.

The perspective of the social ecology model highlights the interrelatedness between people and their surrounding systems [71]. Environmental factors and experiences in social environments influence one’s behaviors and experiences. In other words, human behaviors and experiences are the results of the intersection of the influences of multiple determinants in different systems [71]. For school-age adolescents, family and school are two significant social systems, and how they perceive and experience these two social systems, together with their personalities, may work together to influence their developmental outcomes, including academic stress. However, to date, few studies have put factors from different domains into one model to examine how they work together to affect academic stress or investigate what the more significant correlates associated with academic stress are.

### 2.5. Role of Gender in Academic Stress

According to the literature on stress, gender plays a crucial role in predicting stress and stress escalation. Several studies have indicated that girls experience a higher level of academic stress [21,62] and suffer more from psychopathology associated with school stress. However, the findings are not conclusive. Other studies have suggested that gender does not play a significant role in predicting stress and is not associated with academic stress [14,72]. The reasons for such mixed findings may relate to the differences in research focuses, ages of the participants, variables involved, and cultural contexts examined. For example, girls are more likely to adopt maladaptive perfectionism [2,31] and have closer parent–child relationships [73]. These two traits may expose girls to higher levels of academic stress. However, boys tend to endorse social-oriented achievement motivation because they experience higher parental and societal expectations with regard to their academic achievement in Eastern culture [41,74], which may also be linked to greater academic stress. These differences in traits between genders may affect the level of academic stress. To obtain a more comprehensive understanding of how gender influences the connections between academic stress and its correlates, a gender comparison was conducted in this study. 

### 2.6. Aims of the Present Study

In summary, based on the abovementioned review of literature, the present study used quantitative research method to collect data from high school students to examine how different factors from the personal, familial, and school domains work together to predict academic stress in Hong Kong where the level of academic stress among adolescents is high. In addition, this study also examined whether the theoretical model was applicable to both males and females. Such investigation can inform us which factor(s) is the most significant in leading to academic stress, and which sex group is at a higher risk in bearing academic stress. With theoretical knowledge of the differential significance of factors from the different domains and the identification of the high-risk group, we can then develop effective and targeted measures for preventing and combating academic stress for Hong Kong high school students. Grounded on the literature review, we hypothesized that perfectionism, social-oriented achievement motivation, parental aspiration for achievement, parent–child relationship, and emphasis on academics in school have positive and significant associations with academic stress, while school climate is negatively associated with academic stress. In addition, we also hypothesized that there is no significant difference between boys and girls in the associations between these correlates and academic stress. 

## 3. Current Study

### 3.1. Participants

The data used in this study were collected from secondary 4 to 6 (grades 10 to 12) students from eight secondary schools chosen from different districts in Hong Kong. Convenience sampling strategies were used in this study for obtaining the data. Written consent was obtained from school principals, students, and their parents before administering the survey. The questionnaire, procedures, and consent forms were approved by the Internal Review Board of the Chinese University of Hong Kong, which also provided general ethical oversight for the study. After receiving consent from their parents or guardians, a total of 2072 students were invited to participate in the survey. A total of 12.5% (*n* = 258) of the students did not take part, either because consent was not given by the students or their parents or because they were absent from the class during which the survey was conducted. Finally, a total of 1814 students from the senior forms successfully participated. Ten questionnaires were excluded because they were incomplete. As a result, the final data consisted of 1804 entries. Of the sample, 789 (43.8%) were boys, 1012 (56.2%) were girls, and three did not indicate their gender. The grade-level distribution was as follows: 710 (39.4%) students were in secondary 4 (grade 10), 716 (39.7%) students were in secondary 5 (grade 11), and 378 (20.9%) students were in secondary 6 (grade 12).

### 3.2. Procedure

A self-administered anonymous survey was conducted in the classroom under the guidance of the teacher. The students were informed of the background of the study, and they were encouraged to respond truthfully. The questionnaire included approximately 70 items asking about the participants’ demographic information, their opinions on perfectionism and achievement orientation, and their personal experiences in the school and the family. The survey took approximately 20 min to complete. Written consent was obtained from both students and their parents or guardians before the formal survey was administered. They were informed that their participation was entirely voluntary, and they were free to withdraw from the study at any time and for any reason. The questionnaire, the related procedures, the informed consent forms, and compliance with ethical practices were reviewed and supervised by the university with which the author is affiliated.

## 4. Measures

### 4.1. Gender

The students were asked to indicate whether they were male or female.

### 4.2. Demographic Information

The students were asked to report their demographic background on nine items, including the year of education, age, parents’ highest level of educational attainment, occupational background of parents, living arrangements of the family, family financial situation, and their academic ranking in the class in the last semester.

### 4.3. Perfectionism

This variable was measured by six items that asked the students the extent to which they agreed with the descriptions. The items were selected from the Almost Perfect Scale Revised (APS-R) (Slaney et al., 2001) and the Chinese Version of the Multidimensional Perfectionism Scale (CMPS) [75,76,77]. A 5-point Likert scale (“1 = strongly disagree” to “5 = strongly agree”) was used. The results of an exploratory factor analysis suggested that there were two indicators of this latent variable, namely, (1) discrepancy (factor loading = 0.67), sample questions: “I am never satisfied with my performance” and “Even if I have tried my best, I am never satisfied”; and (2) concern over mistakes (factor loading = 0.83), sample questions: “If I failed academically, I am a failure” and “If I made a mistake, others will perceive me as not being a good person.” The Cronbach’s alpha for these six items was 0.762.

### 4.4. Social-Oriented Achievement Motivation

This variable was measured with 10 items assessing students’ endorsement of social-oriented achievement motivation. These items were selected from a scale developed by Yu and Yang [32]. A 5-point Likert scale (“1 = very true for me” to “5 = not at all true for me”) was used. The results of an exploratory factor analysis suggested that there were two indicators from these 10 items: (1) academic motivation to please others (factor loading = 0.69), sample questions: “I will make an effort to achieve what my parents expect me academically” and “the expectations and demands from my teachers drive my studying;” and (2) academic motivation of instrumental gain (factor loading = 0.80), sample questions: “The reason for industriously studying is to have good future prospects” and “I do not care whether I am interested in learning; I only care about my examination results.” The Cronbach’s alpha for these 10 items was 0.839.

### 4.5. Parent–Child Relationship

This variable was measured with four items asking students about their perception of the parent–child relationship. The items were selected from a scale developed in Taiwan [78]. The items were rated on a 4-point Likert scale (“1 = entirely not true for me” to “4 = entirely true for me”). The results of an exploratory factor analysis suggested that the parent–child relationship was unidimensional. To build a robust latent structure for the parent–child relationship, these four items were placed into two parcels. Parcel one (factor loading = 0.84) included two items, including “my parents are willing to listen to me till I have finished my sharing.” Parcel two (factor loading = 0.84) consisted of two items, including “My parents encourage me.” The score for this scale was calculated by summing up these four items, with a higher score indicating a more intimate parent–child relationship. The Cronbach’s alpha for these four items was 0.778.

### 4.6. Parental Aspiration for Achievement

This variable was measured with six items asking how the students perceived their parents’ expectations of their achievements. These six items were selected from a subscale of the “Living up to parental expectations inventory” (LPEI) [19]. The items were all rated on a 6-point Likert scale (“1 = never experienced” to “6 = strongly experienced”). The results of an exploratory factor analysis suggested that the scale was unidimensional. To build a robust latent structure for perceived parental achievement pressure, these six items were randomly placed into three parcels. The first parcel included two items, including “my parents expect me to choose a subject that they like” (factor loading for this parcel = 0.87). The second parcel included two other items, including “my parents expect my academic attainment to make them proud” (factor loading for this second parcel = 0.92). The third parcel also consisted of two items, including “My parents expect me to have a better academic performance than other people” (factor loading for this third parcel = 0.84). The score for this scale was calculated by summing these six items, with a higher score indicating a perception of greater parental achievement pressure. The Cronbach’s alpha for these six items was 0.892.

### 4.7. School Climate

This variable was measured with seven items asking how the students perceived the school climate. The items were translated from the “Inventory of school climate-student ISC-S” [64]. These seven items were rated on a 4-point Likert scale (“1 = totally disagree” to “4 = totally agree”). The results of an exploratory factor analysis indicated that there were two indicators of the variable: (1) study atmosphere, which comprised four items, including “students work hard for good grades in class” and “grades are very important to students” (factor loading for study atmosphere = 0.75), and (2) positive peer interactions, which comprised three items, including “Students get to know each other well in classes” and “Students enjoy doing things with each other during school activities” (factor loading for positive peer interactions = 0.32). The Cronbach’s alpha for these seven items was 0.767.

### 4.8. Emphasis on Academics in School

This variable was measured with three items asking students about the extent to which they would agree with the descriptions of the school emphasis on academics. The three items were adopted from the study “The nature of social practice among school professionals: consequences of the academic pressure exerted by teachers in their teaching,” which was conducted in Norway [68]. The questions were rated on a 4-point scale (“1 = totally disagree” to “4 = totally agree”). The three items were “This school sets high standards for pupil performance” (factor loading = 0.77), “This school pushes students so that they perform better” (factor loading = 0.82), and “This school makes sure that students are always confronted with challenges” (factor loading = 0.64). The Cronbach’s alpha for these three items was 0.784.

### 4.9. Academic Stress

Seventeen items were used to assess academic stress. These items asked students their actual feelings regarding different descriptions. The items were rated on a 5-point Likert scale (“1 = totally disagree” to “5 = totally agree”). This latent variable consisted of four subscales with a total of 17 items. The first three subscales were academic expectations from parents (five items, factor loading = 0.79), academic expectations from teachers (three items, factor loading = 0.74), and academic expectations from students themselves (six items, factor loading = 0.81). The fourth subscale was excessive demands (three items, factor loading = 0.45). All 17 items were selected from the inventories in three research studies [78,79,80]. The following are sample questions: “I will blame myself if I cannot satisfy my parents’ academic expectations” (academic expectation from parents), “If I have a poor performance in school, I think they are disappointed in me” (academic expectation from teachers), “If I cannot meet my own expectations, I am not good enough” (self-academic expectations), and “The assessments and examinations are too much for me, and I feel that they are unbearable” (excessive demands). The score for this scale was calculated by summing these 17 items, with a higher score indicating a higher level of academic stress. The Cronbach’s alpha for these items was 0.919.

### 4.10. Plan of Analysis

Descriptive statistics, such as the means, standard deviations, and correlations, were calculated for the variables in this study using Statistical Package for Social Sciences (SPSS) 27. The primary analysis method in this study was latent variable structural equation modeling (SEM) with maximum likelihood estimation using the AMOS program (version 27). Confirmatory factor analysis (CFA) was first conducted to ensure that the measurement model had a good fit [81]. Next, the SEM was tested with the full dataset. Cross-group comparison analyses were further conducted to determine if the model could be applied to both males and females. In this comparative analysis, all the factor loadings and the paths of the same model were constrained to be simultaneously equal across genders. Then, the model was tested by releasing the path constraints to determine whether releasing the equality constraint could significantly improve the fit. The normed fit index (NFI), comparative fit index (CFI), incremental fit index (IFI), and root mean square error of approximation (RMSEA) are typically recommended as indicators of the goodness of fit to assess the size of discrepancies between the data and the theoretical model. Typically, an NFI, CFI, and IFI above 0.95 and an RMSEA below 0.06 indicate that the model fits the data well [82,83,84]. A number of demographic variables were added to the model as control variables before conducting the SEM analysis. They were family economic status, father’s education level, and mother’s education level.

## 5. Results

### 5.1. Descriptive Statistics

Table 1 lists the descriptive statistics (means and standard deviations) of the study variables broken down by gender. Table 2 shows the correlations among the seven variables. All of the variables were positively associated with each other, with the exception of two that were both related to the parent–child relationship, with correlations identified between the parent–child relationship and perfectionism (*r* = −0.049, *p* < 0.05) and between the parent–child relationship and parental aspiration for achievement (*r* = −0.132, *p* < 0.01). Both correlations were negative and relatively weak, with the former being the weakest among all the correlations. The two strongest associations were related to academic stress, namely, between academic stress and perfectionism (*r* = 0.536, *p* < 0.01) and between academic stress and social-oriented achievement motivation (*r* = 0.526, *p* < 0.01). The other two relatively strong associations were related to social-oriented achievement motivation, namely, between social-oriented achievement motivation and perfectionism (*r* = 0.444, *p* < 0.01) and between social-oriented achievement motivation and parental aspiration for achievement (*r* = 0.428, *p* < 0.01). Moderate associations were also found for academic stress, namely, between academic stress and parental aspiration for achievement (*r* = 0.325, *p* < 0.01) and between academic stress and emphasis on academics in school (*r* = 0.263, *p* < 0.01). The other moderate association was between perfectionism and parental aspiration for achievement (*r* = 0.275, *p* < 0.01). All the remaining correlations, including the two negative correlations, were relatively weak.

### 5.2. Overall Model

The results of the analysis based on the total sample provided a good fit to the data [χ^2^ (167, *N* = 1804) = 1532.98, *p* < 0.001, with NFI = 0.898, CFI = 0.908, IFI = 0.908, and RMSEA = 0.067].

**Figure 1:**
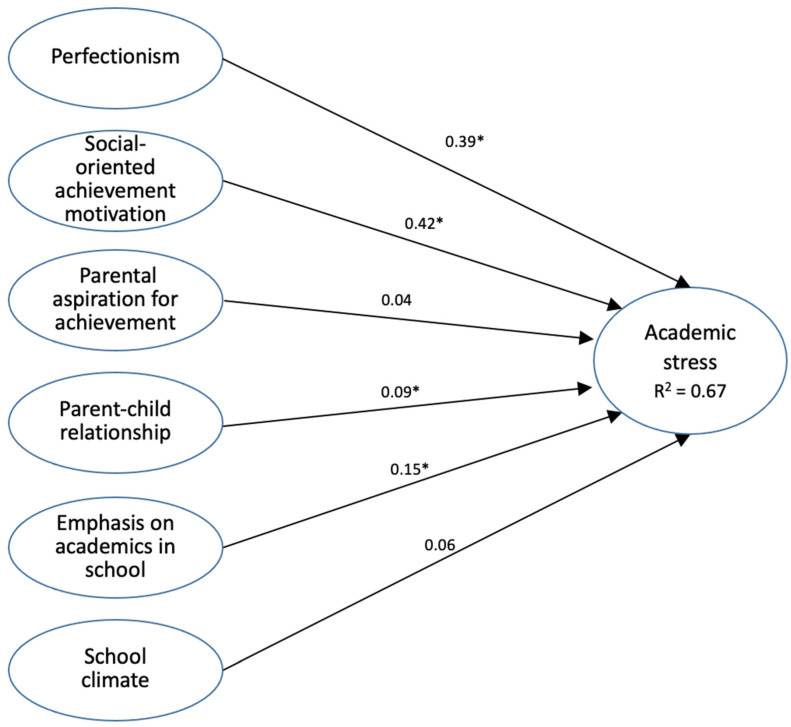
Structural equation modelling of effects on academic stress by perfectionism, social-oriented achievement motivation, parental aspiration for achievement, parent–child relationship, emphasis on academics in school, and school climate (numbers are the standardized effects. Those with * are significant at *p* < 0.01).

Figure 1 shows the paths in the overall model. Among the six paths to academic stress, two were not significant: that from parental aspiration for achievement (*β* = 0.04, *p* = 0.138) and that from school climate (*β* = 0.06, *p* = 0.046). The other four paths indicate that there were significant relationships between the independent variables and academic stress, as follows: social-oriented achievement motivation (*β* = 0.42, *p* < 0.01), perfectionism (*β* = 0.39, *p* < 0.01), emphasis on academics in school (*β* = 0.15, *p* < 0.01), and parent–child relationship (*β* = 0.09, *p* < 0.01).

Overall, all these variables accounted for 67% of the explained variance in the dependent variable academic stress (R^2^ = 0.67).

### 5.3. Gender Comparison

In this analysis, the paths in the same model were constrained to be simultaneously equal in the male and female subgroups. The analysis showed that there was a good fit to the data [χ^2^ (382, *N*: males = 789, females = 1012) = 1851.34, *p* < 0.001, with NFI = 0.880, CFI = 0.901, IFI = 0.902, and RMSEA = 0.046].

**Figure 2:**
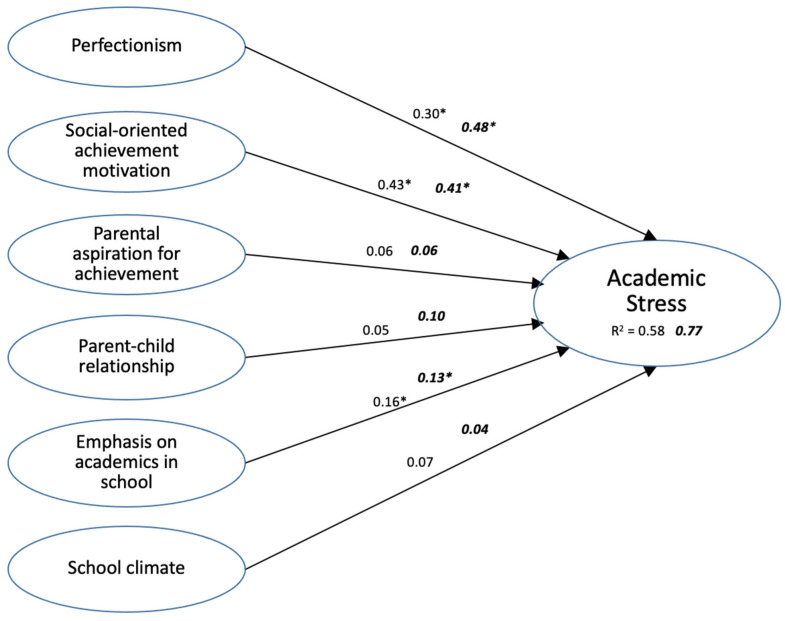
Structural equation modelling of effects on males’ (regular print) and females’ (bold italics) academic stress by perfectionism, social-oriented achievement motivation, parental aspiration for achievement, parent–child relationship, emphasis on academics in school, and school climate (numbers are the standardized effects. Those with * are significant at *p* < 0.01).

Next, the model was tested to determine whether releasing the constraints on the six paths could significantly improve the fit. After releasing the six path constraints, the change in the CMIN with 6 degrees of freedom (df) was 4.2, which was less than the critical value (12.692, *p* < 0.05), indicating that statistically there was no difference in the model between the male and female subgroups (Figure 2).

## 6. Discussion

Unlike most other studies on academic stress, which examined the associations of individual factors with academic stress separately, the present study is one of few studies incorporating different factors from personal, familial, and school domains and examining how these factors work together to predict academic stress in the East Asian context. In addition, the present study also compared the fit of the model between genders to assess whether the model is applicable to both males and females.

Perfectionism, endorsement of social-oriented achievement motivation, parental aspiration for achievement, parent–child relationship, school climate, and emphasis on academics in school were entered into structural equation modelling to examine the most important factors associated with academic stress, and four variables emerged as significant predictors of academic stress. This study found that students who have maladaptive perfectionism are more likely to report experiencing academic stress [2]. The findings are consistent with previous studies on the association of stress with maladaptive perfectionism. This result suggests that students who are very concerned about making mistakes and have a persistent sense of inadequacy with regard to meeting expectations are particularly vulnerable to experiencing academic stress. On the other hand, the results of this study show that students who endorse social-oriented achievement motivation are more likely to experience academic stress. This empirical evidence supports the findings of previous studies that reported a positive association between endorsing social-oriented achievement motivation and experiencing examination stress [13]. The findings of the present study indicate that the urge to please significant others and the drive to obtain instrumental gain make academic achievements very important; if such achievement is beyond the capacity of the students, academic stress is likely to occur. Similar to other studies conducted in East Asian societies, the present research shows that there is a significant positive association between the parent–child relationship and academic stress, i.e., the closer the parent–child relationship is, the greater the academic stress. This result suggests that a closer parental relationship could lead to an urge to meet parental academic expectations. As a result, the pressurized need to attain academic success becomes a cause of substantial academic stress [18,48]. It is noteworthy that the findings of the present study are contradictory to the results of studies conducted in Western societies, which have suggested that intimate parent–child relationships could protect children from stress [44,45]. Last but not least, the findings of this study reveal that emphasis on academics in school has a significant positive association with academic stress. The results indicate that when students’ perceived academic achievement is strongly emphasized in schools, the students are more likely to experience academic stress. This result is consistent with the findings of previous studies [62,85], which have suggested that students in a highly demanding learning atmosphere are more vulnerable to suffering from academic stress.

Two variables were not associated with academic stress, which is contrary to our hypothesis. The results of this study show that parental aspiration for achievement is not a significant predictor of academic stress in Hong Kong. This finding is in disagreement with those of most previous studies, which have found that perceived parental aspiration for achievement triggers greater academic stress or examination anxiety among adolescents [54,86]. It may be that the association between parental aspiration for achievement and academic stress is indirect and depends on certain psychosocial mechanisms, such as optimism [42], or other mediating factors from the family system, such as parental overinvolvement in academic affairs [55]. Further studies might confirm these propositions. In addition, the results of this study show that there is no significant association between school climate and academic stress. This finding contradicts previous theories and studies, which have proposed that the school atmosphere and interactions among students are crucial factors for helping students avoid academic stress [23,65]. Perhaps the nonsignificant association can be attributed to the different dimensions of school climate selected for investigation. In the present study, the school climate comprised two factors, i.e., study atmosphere and positive peer interactions. In future studies, the dimensions of interactions with teachers and the positive motivation received from teachers could be included and explored. In addition, the association between school climate and academic stress may also be indirect and dependent on certain psychosocial mechanisms, such as school connectedness [24]. Further investigations might confirm these propositions.

With regard to gender differences, the results of this study show that the same theoretical model fits equally well for both genders, signifying that the associations of academic stress with the six factors from the personal, familial, and school domains are similar for males and females.

## 7. Implications for Theories, Policies, and Practices

### 7.1. Implications for Theories

Regarding the significant predictors in this model, the two personal factors account for the greatest effect. Different theories propose different factors that are crucial for predicting the stress level, and the association between these individual factors and academic stress is supported in separate empirical studies. However, few empirical studies have incorporated factors from the three domains into one model to examine how the interactions among these factors affect academic stress. The present study was conducted to fill this research gap and provide empirical evidence to support the theoretical arguments. The findings of the present study indicate that the two factors from the personal domain, i.e., perfectionism and social-oriented achievement motivation, have the strongest significant association with academic stress when compared with the effect sizes of the other factors from the family and school domains. These results, to a certain extent, support the proposition that individual personality plays a more crucial role in determining one’s level of stress [27,87]. Although some other studies have argued that family and school experiences have important influences on stress [23,24,55,88], the impacts of those factors from the family and school domains are far less critical than those of personal factors, as shown in the present study. Nevertheless, it is important to note that the examination of the differential impacts of the factors from these three domains was based on the interactions among six factors that were specifically chosen for this study in the East Asian context. Further investigation is necessary to confirm this proposition by incorporating different factors and conducting research in different cultural contexts.

In East Asian societies, the aspiration to achieve excellence in academic performance is embedded in cultural values [52] and is promoted in family and school, the two significant social systems for school-aged adolescents. As a result, students in East Asian societies are likely to be susceptible to stress due to their perception of academic pressure imposed by their significant others [48,89]. Based on these viewpoints, the current study hypothesized that parental aspirations for achievement and emphasis in academics in school are both predictors of academic stress. Nevertheless, the findings of the present study indicate that only school emphasis on academics was significantly associated with academic stress, while the perception of parental aspirations for achievement was not. The results indicate that students become more vulnerable when they perceive pressure due to the academic demands made by schools. One of the reasons for this may be that the pressure from schools could be conveyed through frequent assessments of academic performance, such as daily schoolwork, regular tests, or oral reminders in lessons [23,65,90]. The demanding academic atmosphere in schools may then become a source of stress for the students, particularly when the students are facing high-stakes examinations, the results of which largely determine their future trajectory. To confirm this speculation, future research is needed to examine the frequency and intensity of academic monitoring as mediators of the association between school emphasis on academics and academic stress. For the nonsignificant association between parental aspirations for achievement and academic stress, the result is in contrast with our proposition on how parenting in East Asian societies affects adolescents’ academic achievement and adjustment. It is probable that the association is subject to other factors or psychosocial mechanisms. In East Asian culture, interdependence with family members is highly emphasized; pursuing academic goals and overcoming failure are then not only personal projects but also a team effort in the family [91]. As a result, parents may be actively involved in finishing academic assignments with their children to the extent that such overinvolvement may trigger academic stress. In other words, parental aspiration for achievement may influence the level of academic stress through certain parenting practices, such as overinvolvement and close monitoring on academic matters, which is described as a tiger mother parenting approach. To confirm such a proposition, future studies may include parenting practice as an extraneous variable for re-examining the association between parental aspirations for achievement and academic stress.

### 7.2. Implications for Policies and Social Work Practices

In Hong Kong, although some surveys have indicated that parental academic expectations and school contexts are leading causes of academic stress [92,93], the findings of the present study indicate that personal factors are the major explanatory variables. In addition to personal factors, the present study also suggests that academic pressure from schools plays a crucial role in inducing academic stress. These results can be used to guide resource allocation when attempting to alleviate academic stress. Specifically, these findings suggest that policymakers should design appropriate campaigns to raise awareness of the potential threats posed by maladaptive perfectionism and the endorsement of social-oriented achievement motivation. In addition, the Education Bureau of Hong Kong Government, which is responsible for planning education policies in Hong Kong, may consider strategies to reduce students’ academic pressure by reviewing learning curriculum and the frequency of public examinations. Professional teacher organizations in Hong Kong could also consider comprehensive strategies to alleviate academic pressure by relaxing the highly demanding academic atmosphere and promoting supportive learning culture in schools. 

The major findings of the present study suggest that students who have maladaptive perfectionism and endorse social-oriented achievement motivation are more likely to report experiencing academic stress. Hence, cognitive behavioral therapy may be a useful approach for clinicians to take to help students with perfectionism by identifying and disputing destructive and irrational thought patterns, such that it is imperative to obtain a good result and that their worth is connected to their academic success. The aim of psychotherapeutic treatment for students with maladaptive perfectionism is to encourage them to have broader and more flexible views on success and achievement. In addition, cognitive restructuring interventions can help students who have endorsed social-oriented achievement motivation to reexamine their academic goals and their objectives with regard to academic achievement. Social work practitioners can encourage students to set their own goals and decide their educational paths based on their personal interests and their abilities rather than merely seeking to please others.

## 8. Limitations

Some limitations should be considered when interpreting the results of this study. First, this research was based on cross-sectional data; thus, the findings cannot be used to determine any causal relationship. In addition, the non-significant associations between academic stress and the two factors, i.e., school climate and parental aspiration for achievement, may be due to the research method chosen in this study. Future studies may adopt alternative research methods, such as case study or longitudinal panel design to investigate the associations among the variables in this study. Second, the data collected for perfectionism, social-oriented achievement motivation, parent–child relationship, parental aspiration for achievement, school climate, emphasis on academics in school, and academic stress were self-reported by students, who might have exaggerated their perceptions or underreported their level of academic stress due to social desirability bias and the sensitivity of the issue of mental distress. Data from multiple informants, such as parents or teachers, could be considered in the future to support the theoretical model. Third, the questionnaires were distributed by classroom teachers, and those teachers were also responsible for explaining the background of the study and the instructions for filling out the questionnaire. The messages might not have been conveyed in a clear and unified way, even though the teachers were debriefed. Fourth, despite the large sample size, this study used a convenience sampling of students in Hong Kong. Hence, the conclusions should be interpreted with caution and should not be generalized to other age groups or cultural contexts. Finally, the data were collected during the coronavirus 2019 (COVID-19) pandemic, when the academic schedule in Hong Kong was considerably different. Students experienced uncertainty with regard to their schooling and the arrangement of public examinations, which may have affected their experiences in school and their level of academic stress. Hence, the results of this study should be interpreted with caution.

## 9. Conclusions

In sum, the present study provides quantitative findings of how different factors from the personal, familial, and school domains interact to generate academic stress in the East Asian context. The findings indicate that two factors from the personal domain, i.e., perfectionism and endorsement of social-oriented achievement motivation, are the dominant drivers of academic stress. In addition, the results also show that the patterns of associations and significance of the factors are similar across genders. This similarity implies that the same policies and social work intervention programs could be implemented for both genders.

## Figures and Tables

**Table 1:** Descriptive statistics of the research variables.

Means and Standard Deviations for Each Scale (Standard Deviations in Parentheses)			
	Overall	Gender	
		Male	Female
Perfectionism ^a^	20.33 (4.45)	19.98 (4.31)	20.60 (4.54)
Social-oriented achievement motivation ^b^	20.27 (4.81)	19.89 (4.77)	20.57 (4.82)
Parental aspiration for achievement ^c^	19.03 (6.56)	19.30 (6.34)	18.80 (6.71)
Parent–child relationship ^d^	11.43 (2.24)	11.08 (2.29)	11.70 (2.17)
Emphasis on academics in school ^e^	8.95 (1.40)	8.84 (1.42)	9.03 (1.38)
School climate ^e^	20.71 (2.50)	20.61 (2.59)	20.78 (2.43)
Academic stress ^a^	57.76 (11.19)	55.14 (11.62)	59.80 (10.41)

^a^ On a scale: from 1 = “Totally disagree” to 5 = “Totally agree”. ^b^ On a scale: from 1 = “Very true for me” to 5 = “Not at all true for me”. ^c^ On a scale: from 1 = “Never experienced” to 6 = “Very strongly experienced”. ^d^ On a scale: from 1 = “Entirely not true for me” to 4 = “Entirely true for me”. ^e^ On a scale: from 1 = “Totally disagree” to 4 = “Totally agree”.

**Table 2:** Intercorrelations between variables in this study.

	1	2	3	4	5	6	7
Perfectionism		0.444 **	0.275 **	−0.049 *	0.114 **	0.059 *	0.536 **
2. Social-oriented achievement motivation			0.428 **	0.226 **	0.064 **	0.156 **	0.526 **
3. Parental aspiration for achievement				−0.132 **	0.066 **	0.105 **	0.325 **
4. Parent–child relationship					0.082 **	0.165 **	0.112 **
5. Emphasis on academics in school						0.234 **	0.263 **
6. School climate							0.156 **
7. Academic stress							

** *p* < 0.01; * *p* < 0.05.

## Data Availability

The data presented in this study are available on request from the corresponding author and with permission of the Survey and Behavioural Research Ethnic Committee, The Chinese University of Hong Kong.
